# Large-scale delivery of seasonal malaria chemoprevention to children under 10 in Senegal: an economic analysis

**DOI:** 10.1093/heapol/czx084

**Published:** 2017-07-24

**Authors:** Catherine Pitt, Mouhamed Ndiaye, Lesong Conteh, Ousmane Sy, El Hadj Ba, Badara Cissé, Jules F Gomis, Oumar Gaye, Jean-Louis Ndiaye, Paul J Milligan

**Affiliations:** 1 Department of Global Health & Development, London School of Hygiene & Tropical Medicine, London, UK,; 2 Department of Parasitology, Université Cheikh Anta Diop, Dakar, Senegal,; 3 Health Economics Group, Department of Infectious Disease Epidemiology, School of Public Health, Imperial College London, London, UK,; 4 Institut de Recherche pour le Développement, Dakar, Senegal and; 5 Department of Infectious Disease Epidemiology, London School of Hygiene & Tropical Medicine, London, UK

**Keywords:** Seasonal malaria chemoprevention (SMC), intermittent preventive treatment, malaria, cost function, cost variation, primary health care, community health workers, mass drug administration, campaigns, Sub-Saharan Africa

## Abstract

Seasonal Malaria Chemoprevention (SMC) is recommended for children under 5 in the Sahel and sub-Sahel. The burden in older children may justify extending the age range, as has been done effectively in Senegal. We examine costs of door-to-door SMC delivery to children up to 10 years by community health workers (CHWs). We analysed incremental financial and economic costs at district level and below from a health service perspective. We examined project accounts and prospectively collected data from 405 CHWs, 46 health posts, and 4 district headquarters by introducing questionnaires in advance and completing them after each monthly implementation round. Affordability was explored by comparing financial costs of SMC to relevant existing health expenditure levels. Costs were disaggregated by administration month and by health service level. We used linear regression models to identify factors associated with cost variation between health posts. The financial cost to administer SMC to 180 000 children over one malaria season, reaching ∼93% of children with all three intended courses of SMC was $234 549 (constant 2010 USD) or $0.50 per monthly course administered. Excluding research–participation incentives, the financial cost was $0.32 per resident (all ages) in the catchment area, which is 1.2% of Senegal’s general government expenditure on health per capita. Economic costs were 18.7% higher than financial costs at $278 922 or $0.59 per course administered and varied widely between health posts, from $0.38 to $2.74 per course administered. Substantial economies of scale across health posts were found, with the smallest health posts incurring highest average costs per monthly course administered. SMC for children up to 10 is likely to be affordable, particularly where it averts substantial curative care costs. Estimates of likely costs and cost-effectiveness of SMC in other contexts must account for variation in average costs across delivery months and health posts.

Key Messages

- Our estimates of the costs of SMC are lower than previous studies, which may be attributed to the wider age range (0–10 vs 0–5 years), much larger overall scale of delivery, and more limited involvement of researchers in implementation in our study.
- We observed substantial economies of scale in the size of the catchment area of health posts; the average cost curve was L-shaped, consistent with the limited existing empirical literature on provider costs.
- The financial costs of providing SMC for children under ten represent 12% of combined government and international spending on malaria in Senegal and 1.2% of Senegal's general government expenditure on health per capita, making SMC potentially affordable especially if it were to avert substantial curative care costs.

## Introduction

In 2012, the World Health Organization (WHO) recommended Seasonal Malaria Chemoprevention (SMC) for children under 5 living in areas of the Sahel and sub-Sahel with highly seasonal malaria transmission (World Health Organization 2012). Previously known as intermittent preventive treatment of malaria in children (IPTc), SMC consists of providing a treatment dose of an effective antimalarial on a monthly basis for three or four consecutive months of the year in order to maintain therapeutic levels of antimalarial drugs during the period of greatest malaria risk. SMC with sulfadoxine pyrimethamine (SP) plus amodiaquine (AQ) is a highly efficacious intervention, which clinical trials showed to reduce the incidence of malaria by 75% or more amongst children under five who received it in areas of highly seasonal transmission (Bojang *et al.* 2011; Dicko *et al.* 2011; Konate *et al.* 2011; Sinclair *et al.* 2011; Wilson 2011). A large-scale, stepped-wedge, cluster-randomized trial in Senegal found that delivering SMC to children under 10 reduced malaria incidence by 60% (95% CI 54–64) amongst children under 10 and by 26% (95% CI 18–33) in adults and children older than 10 who did not receive SMC, through indirect effects (Cisse *et al.* 2016). Preliminary findings regarding feasibility, safety, effectiveness and costs from this large-scale study were requested and reviewed by WHO’s Technical Expert Group on Preventive Chemotherapy in 2011 (World Health Organization/Global Malaria Program Technical Expert Group on Preventive Chemotherapy 2011), which subsequently recommended SMC.

Since the WHO recommendation, 12 countries have begun delivering SMC and 7 of these countries have been supported by a $67 m grant from UNITAID to expand access to SMC (Malaria Consortium 2016). While 11 of these countries provide SMC for children up to 5 years of age in accordance with the WHO recommendation, on the basis of the large-scale study findings, Senegal’s policy since 2013 has been to provide SMC to children up to age 10. Policy makers and programme managers in other Sahel countries are considering whether to extend the recommended age range for SMC to address the increasing proportion of the malaria burden falling on older children.

As SMC requires repeated contacts with the health system outside the existing schedule of vaccinations and health campaigns, the feasibility and cost of reaching children in rural areas on a large scale have been important factors in deliberations on SMC (World Health Organization/Global Malaria Program Technical Expert Group on Preventive Chemotherapy 2011). Studies which examined potential delivery strategies concluded that community health workers (CHWs) need to play an important role in implementation (Kweku *et al.* 2009; Bojang *et al.* 2011; Patouillard *et al.* 2011). Economic evaluations were conducted alongside several SMC studies to explore which drug combinations and delivery strategies were most cost-effective (Conteh *et al.* 2010; Bojang *et al.* 2011; Patouillard *et al.* 2011), but these were relatively small-scale trials, which may overestimate both the costs and feasibility of implementing SMC at scale. Nonvignon *et al*. (2016) also examined the cost-effectiveness of SMC implementation with intensified household visits. As all economic evaluations published to date have been conducted amongst younger children, however, they do not directly address questions about whether to extend the recommended age range.

We provide an economic analysis of the costs of administering three monthly courses of SMC in 2010 to a population of over 180 000 children aged 3 months to 10 years in central Senegal in the context of the step-wedge trial previously described (Cisse *et al.* 2016). >93% of children in the target age range received all three intended monthly courses of treatment (Ba *et al.* 2017); delivery was highly equitable (Ba *et al.* 2017) and safe (N’Diaye *et al.* 2016) and reduced the prevalence of molecular markers of resistance to SMC drugs (Cisse *et al.* 2016). Extending the preliminary findings reviewed by WHO, we provide a comprehensive analysis of cost drivers, the distribution of costs across the 3 months of administration and across health system levels, variation in costs between health posts, and economies of scale. We aim to inform decisions on whether to extend the recommended age range for SMC and draw conclusions of wider relevance to the implementation of other large-scale health campaigns and the organization of the health system.

## Methods

### Study setting and design

Details of the step-wedge study design (Cisse *et al.* 2016) and study setting (Ba *et al.* 2017) are provided elsewhere. In brief, following two seasons of piloting in a neighbouring district, 54 rural and semi-urban public health post catchment areas in 4 districts (Bambey, Mbour, Fatick and Niakhar) were randomized to start implementing SMC in 2008 (9 catchment areas), 2009 (18 catchment areas) or 2010 (18 catchment areas). Cost data were collected in 2010, when 45 catchment areas (comprising 45 public health posts and 1 mission facility) implemented SMC.

Senegal was classified as a low-income country until 2011, and was reclassified as such in 2017 (World Bank 2017). In the implementation area, 32% of the population was under 10 years old (Cisse *et al.* 2016). The area’s rainy season runs from July to early October and the climate is sudano-sahelian, leading to highly seasonal transmission. While the malaria burden had been very high, it had fallen by 2010, when malaria incidence in the study's control areas (confirmed by a rapid diagnostic test, RDT) was 4.3 cases per thousand children under 5 and 10.0 cases per thousand children aged 5–10 (Cisse *et al.* 2016). In 2014, malaria continued to account for 5.0% of deaths in children under 5 and 3.4% of all deaths in Senegal (PNLP 2015).

### SMC delivery strategy

The existing CHW network, which already delivered a variety of interventions including twice yearly Vitamin A and anthelminthic tablets for children under 5 through door-to-door strategies, was identified as most appropriate for SMC distribution. Under the supervision of the head nurse at each health post, CHWs travelled door-to-door on designated days in September, October, and November to administer the first dose of loose, crushable AQ and SP tablets each month to children aged 3–119 months and to provide AQ tablets for the child’s caregiver to administer using the household’s usual water supply on the subsequent 2 days. In 2010, implementation was organized primarily by the district health management teams (DHMTs).

The head nurse at each health post trained CHWs over the course of several hours on the day before administration in September, but did not repeat this full training in October and November. For CHWs who missed this initial training, informal, on-the-job training was provided, as is standard practice for campaigns. Each head nurse was responsible for organizing the hiring of CHWs and deciding the number of days they were hired for and their payment. Health posts received a lump sum to cover CHW incentive payments, based on the estimated number of CHWs needed and the estimated number of days work it would take the CHWs to cover the target population of the health post. Some nurses chose to divide the lump sum by the number of CHWs associated with their health post and pay CHWs a fixed amount, while others paid a daily rate (Ba *et al.* 2017).

### Perspective and hierarchical boundaries

Detailed data on resource use associated with delivery of SMC were collected to estimate the incremental costs of implementing SMC at scale in 2010. All 45 government health posts that delivered SMC in 2010 were included, as was one mission health post which managed SMC delivery within a defined portion of the official catchment area of one of the 45 government health posts.

The study takes a health service perspective. The opportunity cost for households to participate in SMC is expected to be low as SMC is delivered door-to-door (Conteh *et al.* 2010).

Both financial and economic costs are included. Incremental financial costs reflect the additional funding needed to pay for the intervention. Incremental economic costs reflect the full value of the additional resources used to implement SMC, including those which did not incur an incremental financial cost to the health service, such as the time required of the district health team and health post staff, and items paid for by CHWs or other organizations. The economic value of individuals’ time was calculated as a fraction of their salaries (including benefits) or, for CHWs, estimated earnings, assuming 220 working days per year and an average 7.5-h working day.

We focus on costs of implementation at the district level and below. Costs incurred only at national level, such as those associated with meetings amongst national-level representatives, are not included because they only concerned research; implementation questions were devolved to district managers. Nearly all the costs of implementation from the district level and below were considered recurrent, meaning that they would have to be repeated for each year of implementation. The only capital costs (resources that last over a year) associated with SMC implementation were those of the research team vehicles, which were used in a few instances to support the distribution of SMC drugs and supervision. Straight line depreciation of the purchase price of vehicles was used with a 5-year expected life of the vehicle (based on local usage) and an assumption of 220 working days per year to estimate the daily economic value of these vehicles, in addition to the financial costs of fuel.

Costs of research activities were generally excluded from the analysis. In two cases, however, costs associated with research activities were very likely to have contributed directly to the success of the administration and so they have been included and described in detail. First, all costs of the demographic surveillance system (DSS) set up to support the trial were excluded, however, some of the DSS fieldworkers and supervisors provided supervisory support on the administration days in September and October and transported some of the drugs; the costs of their time and of drivers and vehicles for these implementation activities have, therefore, been included under supervision and supply chain, respectively. As it is standard practice for districts and health posts to request the support of local organizations such as NGOs or research institutes for health campaigns, these costs are considered incremental economic costs, but not incremental financial costs to the Ministry of Health. Second, health staff at post, district, and regional levels received incentives for participation in the research. These incentives were paid over 12 months and were intended to support participation in research activities such as morbidity surveillance. While it is not anticipated that such incentives would be paid if SMC were implemented outside a research context, these incentives may have contributed to more assiduous implementation of SMC, and so they are also presented as a separate cost category.

### Data collection

Tools were developed to collect data on costs and resource use at four levels: the project, the district, the health post, and the CHW. At the district, health post, and CHW levels, questionnaires were developed, introduced to all district medical officers, head nurses, and CHWs at the SMC planning meetings before administration began in 2010, and refined to incorporate their feedback. Trained fieldworkers collected data from all 4 districts and all 46 health posts following each round of administration in September, October, and November. They also administered questionnaires to a systematic sample of CHWs each month. In total, 405 CHW interviews were conducted, reflecting 48% of the average of 822 CHWs who administered SMC each month, or 13% of the CHW-months of administration. District and health post questionnaires and health post and CHW questionnaires covered similar questions regarding resource use, activities, and payments so that data could be triangulated. In addition, several key informant interviews were conducted with local field coordinators and CHWs to compare the per diems paid to CHWs with what they could otherwise have earned on the SMC administration days.

In November, three health posts in Bambey District combined administration of SMC with administration of Vitamin A and mebendazole. For these three posts, and in some cases for Bambey’s district-level costs, the cost of delivering SMC alone in November was estimated based on the costs incurred in these health posts in October.

### Data management and analysis

Questionnaire data were entered into an MS Access database. Consistency checks were performed to ensure data validity and data were exported to MS Excel for analysis. Data were carefully triangulated between sources to maximize accuracy and avoid double-counting. Costs are presented in United States Dollars (USD) based on the average 2010 exchange rate with the West African Franc (1 USD = 495 XOF(OANDA)).

Costs were summarized according to the categories presented in Table 1. These categories were identified to ensure comparability with previous studies of SMC (Conteh *et al.* 2010; Bojang *et al.* 2011) and to reflect the key cost centres. We present key cost drivers and examine several aspects of the cost structure and cost variation. To facilitate projections of how costs may vary if fewer or more monthly rounds of SMC were implemented, we disaggregated costs by the month in which they were incurred. To facilitate projections of how costs may vary with different scales of delivery and in different areas, we disaggregated costs by the health system level (district, health post, CHW, child) with which they would be expected to vary approximately linearly.

**Table 1.** Description of cost categories

Cost category	Description
SMC drugs	Reflects the cost of SP and AQ tablets supplied by National Pharmacy of Senegal and Kinapharma (Accra, Ghana), respectively and actually used or wasted during SMC administration, including the costs of importation to the Port of Dakar
Drug transport/supply chain	Reflects the cost of transporting drugs from Dakar to the districts (via a local storage site) by the research team, and from the districts to the health posts by district and health post staff. Additional economic costs include the value of time and of vehicles used by the research team, districts, and health posts
Drug administration (CHWs)	Includes the cost of payments of per diems to and transport for CHWs to come to the health post, retrieve drugs and registers, administer drugs to children, and return to the health post to return their reports and remaining drugs on each day of the administration. Additional economic costs include transport costs paid by the CHW and not reimbursed by the health facility
Supervision	Reflects the cost of incentive payments to a head nurse, assistant, and in some cases trainee at each health post; to each district health management team, region, and prefecture to supervise the implementation of SMC and to manage any side effects or refusals; and the costs of any transport used for this supervision. Additional economic costs include the value of time and transport for these health staff as well as the DSS supervisors and fieldworkers for the days on which they helped districts to supervise the administration
Training of CHWs	CHWs attended a single training day at their health post before administration in September. The payment of per diems, as well as the costs of any food or supplies provided or used during the training and any transport paid for by the health post or district are included as financial costs. Additional economic costs include the value of health staff time
Training of head nurses	Head nurses travelled to their district headquarters for a one-day training before administration in September. Costs were incurred for the per diems paid to the head nurses, their transport, and the food and supplies provided. Additional economic costs include the value of participants’ time and of vehicles used
Meetings (evaluation & planning)	Prior to the training, head nurses attended one or more evaluation and planning meetings at their district during which they evaluated results of the SMC implementation in 2009 and outlined plans and budgets for implementation of SMC in 2010. Costs include per diems, transport, and any food or materials provided specifically for the meetings. Meetings were held for head nurses at district level and for district managers in Dakar and at one of the districts
Sensitization	Both districts and health posts arranged activities such as travelling caravans, radio announcements, and community meetings to promote awareness of SMC with regional or local authorities and within the community. Additional economic costs include the value of participants’ time and vehicle use
Drugs for side effects	The costs of the small stock of drugs and medical supplies with which to manage potential adverse events provided to health posts were included regardless of the amount used, as these supplies would need to be provided in future as a precaution. In addition, head posts were reimbursed the cost of treating children whose parents reported side effects, in cases where the head nurse used medications other than those provided
Supplies	Supplies used in the administration included hats, t-shirts, and polo shirts with SMC sensitization messages and the MoH logo; registers of children and other monitoring tools; phone cards, etc. In addition, health posts also purchased some supplies themselves, such as pencils and erasers, to complement those provided by the district. Supplies purchased by CHWs are included as economic, rather than financial costs to the health service
Research participation incentives	Regional medical officers, district medical officers and their deputies, district supervisors, and head nurses all received quarterly incentive payments throughout the year to support research activities such as morbidity surveillance. The entire value of these payments over 12 months to the 3 regions, 4 districts, and 45 health posts that implemented SMC in 2010 are included, as they are likely to have contributed to more assiduous implementation of SMC in September, October, and November. It is not expected that this level of incentive payment would be repeated outside a research context

This table provides a detailed description of the cost categories used in the analysis. Where economic costs are greater than financial costs, the source of additional economic costs are mentioned explicitly.

Costs are analysed with respect to several measures of output described in detail elsewhere (Ba *et al.* 2017) (Table 2). Estimates of the number of monthly courses administered were based on administrative data, which was triangulated from routine data in health post reports and administration registers. Estimates of the number of children in the target age range and all residents in the catchment area were based on the DSS. The number of children receiving SMC at least once and in all 3 months was estimated by applying survey estimates of the proportion of children receiving 0, 1, 2, or 3 courses of SMC to the administrative estimate of the number of monthly courses administered. The latter was a conservative approach yielding lower estimates of the number of children receiving SMC (and thus higher costs per course and per child) than applying survey estimates to the target population estimated in the DSS.

**Table 2.** Financial and economic cost of SMC per output

Denominator	Number	Cost of SMC per output (US$)			
		**Financial**		**Economic**	
		Excluding research incentives	Including research incentives	Excluding research incentives	Including research incentives
Monthly courses administered^a^	471,283	$0.41	$0.50	$0.50	$0.59
Children receiving SMC at least once^b^	157,654	$1.21	$1.49	$1.49	$1.77
Children receiving SMC in all three months^b^ (i.e. "fully adherent")	156,311	$1.22	$1.50	$1.51	$1.78
Children of target age in the catchment area^c^	181,060	$1.06	$1.30	$1.30	$1.54
Residents (all ages) of the catchment area^c^	589,332	$0.32	$0.40	$0.40	$0.47

a Based on administrative data, which was triangulated from routine data in health post reports and administration registers (Ba *et al*. 2017).

b Generated by applying survey estimates of the proportion of children receiving 0, 1, 2, or 3 courses of SMC to the estimate of the number of monthly courses administered based on administrative data (Ba *et al.* 2017).

c Based on the DSS (Ba *et al.* 2017).

Affordability was explored by assessing the annual financial cost of SMC per person (all ages) resident in the catchment area as a proportion of three relevant, existing expenditure levels. These were: (1) average annual expenditure for malaria control and elimination per person at risk in Senegal (which includes both domestic expenditure on malaria prevention and treatment and donor funding earmarked for malaria control) in 2013–2015 (WHO Global Malaria Programme, 2016); (2) Senegal’s general government expenditure on health per capita in 2014 (World Health Organization 2017) and (3) Senegal’s total health expenditure per capita in 2014 (World Health Organization 2017). The most recent expenditure levels available are used to explore affordability relative to current funding, but are presented in constant 2010 USD to allow comparison with our cost estimates.

We sought to identify factors associated with variation between health posts in the average cost of SMC administration per course administered. We analysed average economic costs including research participation incentives with the district-level costs divided equally among the health posts within each district. We hypothesized that the following observed variables could be associated with cost variation across health posts: the number of courses administered (i.e. output or scale), coverage, geography of the catchment area (minimum, average, and maximum distances from health post to catchment villages; catchment area; number of catchment villages), and the number of years of experience (of the head nurse and, separately, at the health post) of delivering SMC (0, 1, or 2). Coverage estimates (the number of courses administered as a proportion of the target) were based on administrative, rather than survey data, because survey-based coverage estimates were not available for each health post (Ba *et al.* 2017). As our observations (health posts) were nested within a small number of clusters (districts), we fit a linear model with fixed effects at the district level (Möhring 2012) to account for this clustering. We used STATA 14. All independent variables were centred. Scatter plots of all pairwise variable combinations were used to assess the linearity of relationships; logarithmic transformations were performed on skewed data and a quadratic term was added for any independent variables exhibiting a curvilinear relationship with costs. Possible interactions between the number of courses administered, coverage, and catchment area were explored. We began with a full model containing all independent variables and interaction terms and sequentially removed the variable from the model with the highest *P*-value. Variables were retained in the model if they contributed to the fit of the model with *P* < 0.05, if removal substantially altered coefficients of other variables in the model, or if they were component variables of retained interaction or quadratic terms. Once a parsimonious model was reached, excluded variables were individually retested. Standard regression diagnostics were performed (Chen *et al.* 2003). Likelihood ratio tests were used to determine whether individual variables and groups of variables improved model fit. We also developed a more parsimonious model based on a more stringent criterion of *P* < 0.0032, which corresponds with a Bayes factor of 20 (Altman and Krzywinski 2017) (Supplementary Materials are available at *HEAPOL* online).

## Results

### Costs and affordability

The financial cost to administer SMC to a population of over 180 000 children aged 3 months to 10 years in four districts of Senegal over one malaria season, reaching an estimated 93% (95% CI 91–96) of children with all three monthly courses of SMC (Ba *et al.* 2017) was $234 549 (Table 3) or $0.50 per monthly course administered (Table 2). The economic costs were 19% higher at $278 922 or $0.59 per course administered. When the value of incentives intended for research is removed, the financial and economic costs fall by $43 424 to $0.41 and $0.50 per course administered, respectively. This financial cost of $0.41 per course administered excluding research participation incentives corresponds to $1.22 per child receiving all three scheduled courses, $1.06 per child of target age in the catchment area, and $0.32 per resident (all ages) in the catchment area (Table 2).

**Table 3.** Total financial and economic costs of SMC

	Financial costs			Economic costs		
	Total costs US$ (2010)	Cost profile		Total Costs US$(2010)	Cost profile	
		Including research incentives (%)	Excluding research incentives (%)		Including research incentives (%)	Excluding research incentives (%)
**TOTAL***including research incentives*	**$234 549**	**100.0**	NA	**$278 922**	**100.0**	NA
**TOTAL***excluding research incentives*	**$191 125**	NA	**100.0**	**$235 498**	NA	**100.0**
SMC drugs (SP+AQ)	$53 010	22.6	27.7	$53 010	19.0	22.5
Drug transport/supply chain	$425	0.2	0.2	$3266	1.2	1.4
Drug administration (CHWs)	$80 651	34.4	42.2	$80 651	28.9	34.2
Supervision	$25 156	10.7	13.2	$57 563	20.6	24.4
Training of CHWs	$6946	3.0	3.6	$8956	3.2	3.8
Training of head nurses	$2283	1.0	1.2	$3813	1.4	1.6
Meetings (evaluation & planning)	$2365	1.0	1.2	$3851	1.4	1.6
Sensitization	$2519	1.1	1.3	$2962	1.1	1.3
Drugs for side effects	$2491	1.1	1.3	$2491	0.9	1.1
Supplies	$15 279	6.5	8.0	$18 935	6.8	8.0
Research participation incentives	$43 424	18.5	NA	$43 424	15.6	NA

This cost per resident represented 1.2% of Senegal’s general government expenditure on health per capita, 0.6% of total health expenditure per capita and 12% of combined government and international spending on malaria in Senegal (Supplementary Table S1 is available at *HEAPOL* online).

### Cost drivers

The main cost drivers were door-to-door drug administration (42% of non-research financial costs) and SMC drugs (28% of non-research financial costs, Table 3). Per diems paid to CHWs accounted for most of the drug administration costs and 41% of total non-research financial costs (Supplementary Table S2 is available at *HEAPOL* online). AQ tablets alone accounted for 21% of non-research financial costs, while SP tablets accounted for 7%. Incentives paid to nurses and district staff for participation in the research study increased the financial costs of the intervention by 23% (from $191 049 to $234 462). While research incentives were intended to support data collection rather than implementation and are not normally provided for comparable distribution campaigns, they may have contributed to the high coverage levels achieved, as on average they represented a 7% increase in head nurses’ annual salaries—or ∼15 days’ pay assuming 220 days worked per year—and a > 10% increase in the salaries of district and regional staff (Supplementary Table S1 is available at *HEAPOL* online). Publicity campaigns and other sensitization activities played an important role in achieving high coverage (Ba *et al.* 2017), although they accounted for only 1% of financial and economic costs.

Economic costs were $43 945 greater than financial costs because they also included the value of the time MoH staff and others spent in meetings, trainings, travel and supervision (74% of the additional costs), as well as the economic value of vehicles used and the cost of supplies paid for by CHWs while implementing the intervention. Key informant interviews revealed that the payments made to CHWs (median: $7.49 per day, range $5.05–16.16) were comparable to or greater than the daily rate of pay for agricultural labour, similar to rates paid by non-governmental organizations (NGOs) for health activities, and somewhat higher than health districts pay for other mass campaigns. While the qualifications and opportunities amongst CHWs varied, many were illiterate and unskilled and others were secondary school students (Ba *et al.* 2017). In addition, distribution tended to begin on weekends to ensure both that families were at home and that CHWs would be available without taking them away from other activities (Ba *et al.* 2017). The economic value of CHW time spent implementing the intervention was therefore considered to be fully reflected in the financial costs of the payments made to them, and so no additional economic costs of CHW time were calculated.

### Time allocation

Most CHWs worked in pairs, delivering SMC to a mean of 46 children per CHW per day at each health post (range 25–78) (Supplementary Table S3 is available at *HEAPOL* online). Some CHWs assisted with administrative duties in the health post for some or all of the administration days and so administered few or no courses directly; hence, for individual CHWs who administered at least one course, the average number of courses administered per day varied more widely, from 2 to 169. Health posts employed from 4 to 68 CHWs and delivery each month took from 2 to 6 days per post. CHWs worked a mean of 7 h per day, but this varied from 1 to 12 h across CHWs and the average number of hours per day per CHW also varied substantially between health posts, from an average of just 4 h per day in one health post to an average of 10 h per day in another (Supplementary Table S3 is available at *HEAPOL* online). CHW time spent on SMC was only moderately (22%) higher in September (mean 665 cumulative CHW-hours per health post) than in each subsequent month (543 h) (Supplementary Table S4 is available at HEAPOL online). Head nurses spent a median of 75 cumulative hours on SMC over the 3 months (range 7–156), more than two-thirds of which was spent on the September round (Supplementary Table S4 is available at HEAPOL online).

**Table 4.** Cost of activities by health system level and month of administration

Level	Financial costs					Economic costs				
	Sept (and earlier)	Oct	Nov	Total costs	Cost profile (%)	Sept (and earlier)	Oct	Nov	Total costs	Cost profile (%)
District	$7019	$4234	$5549	$16 801	8.8	$12 235	$7028	$7010	$26 274	11.2
Post	$22 456	$7311	$8809	$38 576	20.2	$37 521	$14 987	$15 416	$67 924	29.1
CHW	$6920	$16	$9	$6946	3.6	$6920	$16	$9	$6946	3.0
Child	$43 939	$42 506	$42 356	$128 802	67.4	$45 351	$43 632	$43 475	$132 457	56.7
Total	$80 334	$54 067	$56 723	$191 125	100.0	$102 027	$65 664	$65 910	$233 601	100.0
Cost profile (%)	42.0	28.3	29.7	100.0		43.7	28.1	28.2	100.0	

Costs are attributed to the lowest level with which they would be expected to increase linearly. For example, if the number of CHW were doubled, but all else held constant, the CHW-level costs would be expected to double while other levels would remain approximately constant. Similarly, adding an additional month to the campaign would add 28–30% to total costs, assuming that this additional month's campaign was conducted similarly to the October and November campaigns, rather than the September campaign, which incurred additional start-up costs, especially for meetings and trainings. Research participation incentives are excluded.

Across the four districts, district medical officers spent a median 78 h (range 12–116) on SMC, while their deputies spent substantially less time (Supplementary Table S4 is available at HEAPOL online). District supervisors spent a median 209 h per district (range 42–376) on SMC. Supervisors and fieldworkers from the DSS supported supervision in September and October, spending the largest amount of time in the district whose supervisors and senior officers spent the least time on SMC. The additional time district and health post staff spent in September relative to the two subsequent months was largely spent in meetings and trainings at both the district and health post level. Several separate meetings/trainings on SMC were held at the districts involving head nurses in August and September; discussions of SMC did not appear to be combined in other district meetings.

### Cost structure

The first of the three monthly SMC rounds accounted for 42% of financial costs (Table 4). Adding a fourth monthly SMC round would be expected to increase total costs by ∼28–30%, assuming that the additional month’s campaign were conducted similarly to the October and November campaigns.

Two-thirds of financial costs (67%) were expected to vary with the number of children, while 20% were expected to vary with the number of health posts (Table 5). Only 9% of financial costs were expected to vary with the number of districts, while <4% of costs were expected to vary with the number of CHWs. Thus, for example, if a new health post were created to serve half the catchment population of the largest health post in our study, we could assume that the number of districts, CHWs and children in our analysis would remain constant, but the number of health posts (and thus the costs expected to vary with the number of health posts) would increase by 2% (1/46). Holding all else constant, the addition of this new health post could then be projected to increase the total financial costs of SMC implementation by 0.4%.

### Cost variation and economies of scale across health posts

**Figure 1.**
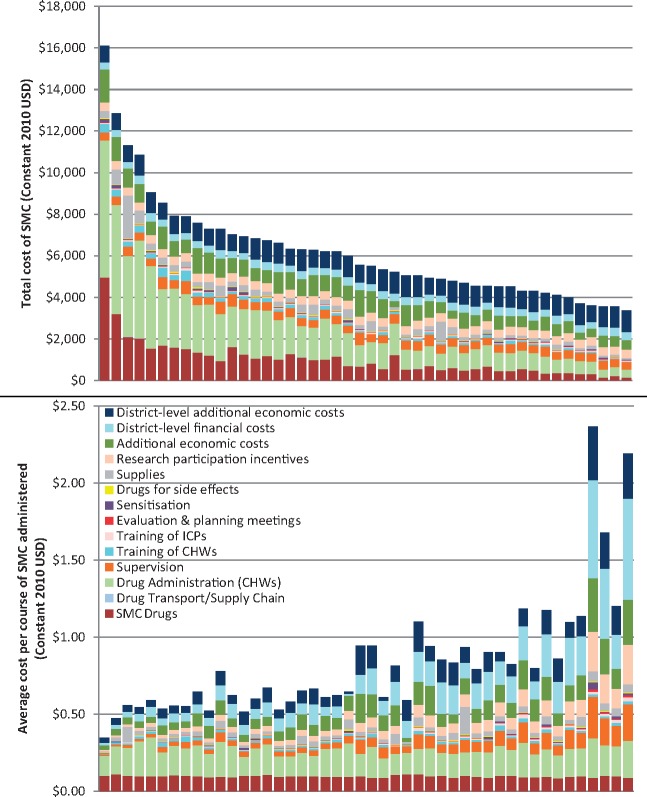
Total and average costs by health post with cost drivers. Health posts are ordered (left to right) in both graphs from largest to smallest total economic costs, including research participation incentives. District-level costs have been divided evenly across the health posts within each district. As total costs decrease, the average cost per course administered tends to increase, although there is some variation in this trend.

**Figure 2.**
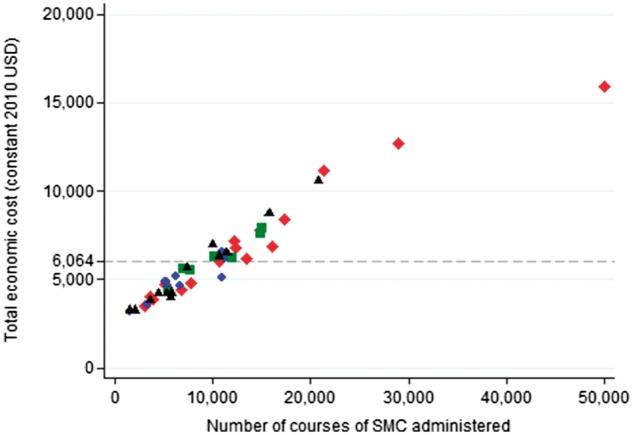
Total economic cost vs the number of courses administered at each health post. The figure illustrates the variation in the total costs incurred for SMC administration between health posts. Costs incurred at the district level are allocated equally across health posts in that district. Research participation incentives paid directly to head nurses and district health staff for trial participation are included as they are likely to have led to more assiduous implementation. The 46 health posts are presented with a different marker for each of the 4 districts. Dashed line: mean total economic cost per health post

The total economic cost varied substantially across the 46 health posts, from $3223 to $15 946 when district-level costs were apportioned equally across health posts within each district and research participation incentives were included (Figures 1 and 2). Costs incurred only at the health post level and below varied from $1558 to $14 573. The average economic cost varied from $0.32 to $2.10 per course administered and from $0.30 to $1.38 when considering only costs incurred at the health post level and below (Supplementary Table S5 is available at *HEAPOL* online). The cost of SMC tablets and the cost of per diems for CHWs were relatively constant with respect to the number of courses administered (Figure 1). In contrast, the remaining significant cost centres, notably supervision, the additional economic value of health worker time, and the research participation incentives, were relatively fixed with respect to the number of health posts and thus account for most of the variation in average cost per course between health posts.

**Figure 3.**
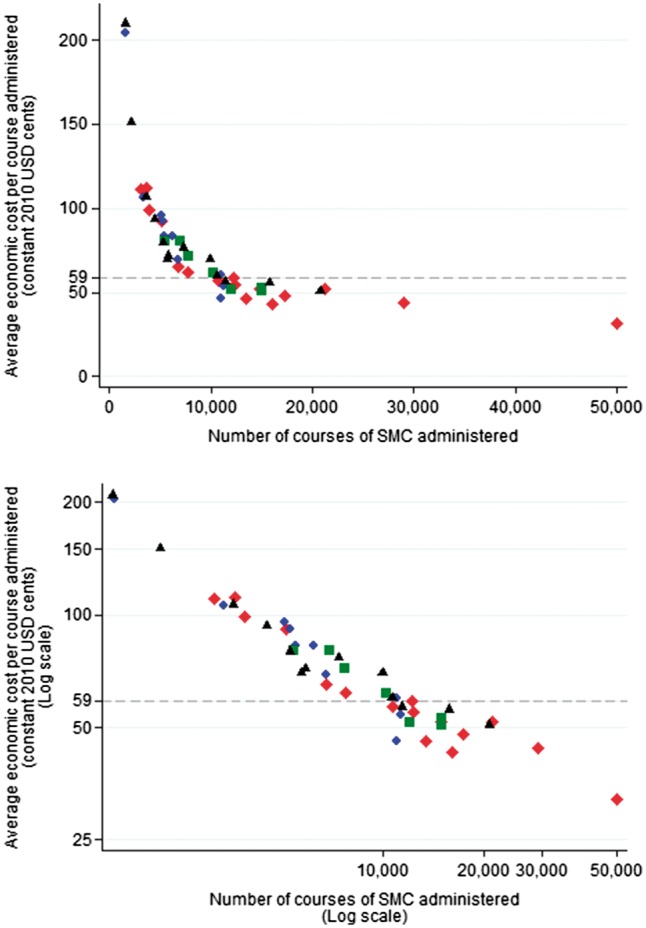
Economies of scale: average economic cost per course administered vs the number of courses administered at each health post. The figure illustrates the variation in the average economic cost per course of SMC administered between health posts. The upper figure presents data on a standard arithmetic scale and the lower figure illustrates the same data with both the x-axis and y-axis presented on a logarithmic scale. Costs incurred at the district level are allocated equally across health posts in that district. Research participation incentives paid directly to head nurses and district health staff for trial participation are included as they are likely to have led to more assiduous implementation. The 46 health posts are presented with a different marker for each of the 4 districts. Dashed line: mean economic cost per course administered across the entire implementation area.

Average costs displayed a strong L-shape when plotted against the number of courses administered (Figure 3). In this sample, there was no evidence of a point at which health posts had such high levels of output that they displayed diseconomies of scale. Average costs increased steeply for health posts administering fewer than ∼8000 courses of SMC (∼10 000 residents), while above 10 000 courses (∼12 500 residents), average costs declined, but more gradually (Figure 3). In exploring factors associated with this variation, we found that using the more stringent, Bayesian criterion of *P* < 0.0032 led to a parsimonious log–log model of average economic costs as a quadratic function of the number of courses administered (including fixed effects at the district level); this model described nearly all the variation in the data (adjusted *R*^2^ = 0.94). The more traditional, frequentist threshold of *P* < 0.05 led to a more complex model (adjusted *R*^2^ = 0.95), which also included variables for the interaction between coverage and size of catchment area and between size of catchment area and the logarithm of the number of courses, as well as the coverage and size of catchment area levels. While the likelihood ratio test indicated that this set of additional variables improved model fit (*P* = 0.020), the large number of variables relative to data points suggests that the more complex model may be overspecified. (Supplementary Table S6 are available at *HEAPOL* online)

## Discussion

From an economic perspective, delivering SMC to children up to 10 years of age appears both affordable and sustainable, even within the highly constrained budgets of West African health systems and before accounting for savings from reductions in malaria cases. A cost-effectiveness analysis would provide further information on the value-for-money of extending the recommended age range for SMC from children under 5 to children under 10 and on decisions regarding where to implement SMC. Two analyses of the cost-effectiveness of SMC in children under 5 have been based on findings from small-scale trials: one trial-based analysis used “cost per case averted” as its outcome metric (Conteh *et al.* 2010), making its findings difficult to compare across health conditions, while a later analysis employing a dynamic transmission model concluded that SMC in children under 5 is likely to be cost-effective or highly cost-effective relative to the arbitrary, but fairly conservative thresholds of $150 and $25 (1993 USD) per disability-adjusted life-year averted (Ross *et al.* 2011). A third analysis estimated the cost per case averted of SMC for children under 5 for a strategy requiring CHWs to visit each household on five consecutive days in each of four consecutive months (Nonvignon 2016). The cost-effectiveness of SMC in children under 10 is likely to be highly dependent on the coverage achieved and the age-specific incidence of malaria, both of which can be expected to vary significantly between countries, between regions within a country, and over time. We have shown that costs also vary substantially across health posts and across distribution rounds; this variation must also be considered in estimating the likely costs and cost-effectiveness of SMC in other settings. In disaggregating SMC costs by the health system level with which they are expected to vary, we offer a preliminary step towards taking cost variation into account in future estimates.

Our estimates are lower than those reported in previous analyses of SMC, which may be attributed to our study’s extended age range, far larger target population, delivery strategy involving only one household visit per month, and more limited involvement of researchers in implementation. Previous studies of SMC examined the costs of delivery only to children up to 5 (or in one case 6) years of age (Conteh *et al.* 2010). In our study, increasing the age range for SMC from under-5s to under-10s virtually doubled the target population, however, it only increased the target number of households to visit by 13%, from 80 to 90% of all households in the area, and high coverage was maintained in both groups (Ba *et al.* 2017). However, the degree to which the lower costs we observed can be attributed to the extended age range rather than the far larger overall target population or other factors remains uncertain. Since our study, co-blister packs of dispersible SP+AQ have become available, which may increase tablet costs while potentially reducing CHW time on administration; these and any other changes to the distribution strategy would need to be accounted for in estimates of the likely costs of SMC in other settings.

While we have focussed on the cost per monthly course of SMC administered as our key outcome measure, other studies of the costs of SMC have reported the cost per “fully adherent child,” defined as a child receiving all three (or more) intended courses of treatment. At $1.50 excluding research incentives (Table 2), the economic cost per fully adherent child in our study was lower than reported estimates from all other studies, and even somewhat lower than projections made of the likely costs of delivering SMC at scale. Inflating costs to 2010 USD to allow some comparison (Bureau of Labor Statistics 2013), the lowest previously reported cost per fully adherent child of a three-course SMC regimen was $1.66 using CHWs to deliver SP and AQ in Basse, Gambia (Bojang *et al.* 2011; Pitt *et al.* 2011). A study in trial conditions in Hohoe, Volta Region, Ghana reported the cost per fully adherent child of delivering three bimonthly courses of SP alone through CHWs at $8.30, but projected a cost of $1.74 if distribution were scaled up to the district level (Conteh *et al.* 2010; Pitt *et al.* 2011). In a wide-ranging overview of malaria control strategies, Goodman *et al.* (2000) estimated the cost of seasonal fortnightly chemoprevention with dapsone and pyrimethamine at $1.79 (90% range $1.40–2.20) using an existing CHW network, but requiring parents to take their children to the health centre. Estimates were substantially higher for the four-course strategies studied in Jasikan, Volta Region, Ghana (Patouillard *et al.* 2011; Pitt *et al.* 2011) and in Ghana's Upper West Region (Nonvignon 2016), and for the three- and six-course strategies employing artesunate (AS) with AQ in Hohoe (Conteh *et al.* 2010; Pitt *et al.* 2011). The “fully adherent child” metric includes the full cost of all the doses of SMC which were administered, but not the benefits derived by children who were protected from malaria with fewer than the intended number of courses of SMC. Given the highly mobile nature of populations both in the study area and in other areas where SMC is likely to be of benefit, children may have missed doses because they were away from the area and therefore either not exposed to malaria or potentially able to receive SMC elsewhere if it were more widely available (Ba *et al.* 2017).

Although the transferability of costs across contexts depends on many factors (Vassall *et al.* 2016), our financial cost estimate of $1.22 per fully adherent child ($1.50 including research participation incentives) is within the range of costs associated with delivering other malaria prevention interventions. A systematic review reported a median financial cost per year of protection with ITNs at $2.20 (range $0.88–9.54, constant 2009 USD), with IRS at $6.70 (range $2.22–12.85), with IPT in infants at $0.60 (range $0.48–1.08), and with IPT in pregnant women at $2.06 (range $0.47–3.36) (White *et al.* 2011). The financial cost of school-based IPT has been reported at $1.20 (constant 2006 USD) per child per year (Temperley *et al.* 2008) and school-based intermittent screening and treatment has been reported to cost $6.61 per child per year (constant 2010 USD) (Drake *et al.* 2011).

Our findings of substantial economies of scale represent an important contribution to a very limited evidence base on cost variation in health service delivery in low- and middle-income countries, particularly at the primary health care and community level and outside the HIV field (Brooker *et al.* 2008; Fiedler *et al.* 2014; Siapka *et al.* 2014). Consistent with previous studies of HIV prevention in India (Guinness *et al.* 2007; Lépine *et al.* 2015; Lépine *et al.* 2016) and of school-based albendazole distribution in Uganda (Brooker *et al.* 2008), we found that average costs exhibited an L-shape, and found no evidence in our sample of a point at which average costs would begin to increase, generating the U-shape predicted in economic theory. While our statistical analysis remains descriptive and was limited by the small number of data points and possibly by the trial context and other factors, the 46 health facilities we analysed constitute a relatively large dataset in the context of health facility costings. Our findings may be particularly relevant for other CHW mass distribution campaigns, such as deworming tablets and vitamin A, and for integrated community case management programmes, for example. They also have wider implications for the organization of the health system; many factors, such as accessibility, must be considered in deciding on the location and catchment size of health facilities, however, our findings demonstrate the substantially higher costs per person reached incurred by health posts with very small catchment areas.

Although appropriate to our study, the incremental nature of our analysis means that the existence of a functioning health system, including a network of CHWs, is assumed. In contexts where, for example, head nurses cannot easily call upon a group of CHWs for distribution campaigns, where the head nurses themselves are absent, or where districts lack the capacity to coordinate training and distribution of incentives, medicines, and materials, additional resources would need to be invested to address or circumvent these gaps. In this way, SMC could provide an opportunity to strengthen health systems and especially CHW networks, but doing so would involve greater costs than reported here.

Furthermore, we have not included the costs of pharmacovigilance, ongoing programme evaluation, and national-level coordination, which are important aspects of SMC implementation and will need to be included in programme budgets. As in all such analyses, our data may also include errors or omissions, however, our extensive triangulation and comparisons across health facilities allowed us to correct discrepancies which might not have otherwise been detected. Nonetheless, collecting data on health worker time use, which accounted for most of the additional economic costs, is particularly challenging, and may have been subject to bias or misreporting.

Finally, while we adhered to standard practice in calculating the economic costs of health worker time based on their salaries, it is very unlikely that any health worker’s salary represents their value to society, especially where they are so exceedingly scarce. Valuable health worker time should be used as efficiently as possible, taking into account the negative effects of nurses frequently absenting themselves from health posts to attend meetings at district level and to supervise CHWs in door-to-door campaigns. Strong national and district-level leadership is therefore required to bring together national child health and disease control teams to limit the total number of off-site training and campaign days each year. Similarly, the availability and supply of CHWs is not infinite. In our context, the value of incentive payments to CHWs were higher than what many CHWs could otherwise have earned, but that does not mean that they would necessarily continue to be willing to implement many more additional campaigns each year. Opportunities may exist to achieve economies of scope by combining SMC with delivery of other interventions, such as mass distribution of ITNs, health communication, or neglected tropical disease programmes; however, careful consideration and discussion with all levels of health workers will be required to ensure that additional interventions are genuinely compatible and do not cause diseconomies of scope by unduly increasing complexity.

## Conclusion

Even in the context of a highly constrained health system, door-to-door delivery of SMC by CHWs to children under ten is likely to be affordable, especially if it averts substantial costs of curative care. We identified substantial variation in the cost of delivering SMC to children in Senegal, which contributes to a very limited evidence base on variation in provider costs. Both cost variation and the comparability of local health system characteristics must be accounted for in assessing the transferability of our findings to other settings.

## Ethics statement

The trial protocol was approved by the Conseil National pour la Recherche en Santé, Senegal and the ethics committee of the London School of Hygiene & Tropical Medicine, UK. The trial is registered at www.clinicaltrials.gov: NCT 00712374.

## Supplementary Material

Supplementary Data
