# Supplementary material for: Large-scale delivery of seasonal malaria chemoprevention to children under 10 in Senegal: an economic analysis
Source: Health Policy Plan. 2017 Jul 24;32(9):1256–66. doi: 10.1093/heapol/czx084 (PMC5886061; doi:10.1093/heapol/czx084)
Supplement: Supplementary Data [file czx084_supplementary_materials.pdf]

SUPPLEMENTARY MATERIALS

Large-scale delivery of seasonal malaria chemoprevention to children under 10 in Senegal: an economic analysis

Catherine Pitt, Mouhamed Ndiaye, Lesong Conteh, Ousmane Sy, El Hadj Ba, Badra Cissé, Jules F Gomis, Oumar Gaye, Jean-Louis Ndiaye and Paul J Milligan

CONTENTS

Supplementary Table S1. SMC costs in context

Supplementary Table S2. Input costs of key cost drivers

Supplementary Table S3. Resources used in SMC delivery

Supplementary Table S4. Variation in health worker time spent on SMC by month and catchment area

Supplementary Table S5. Descriptive statistics: Cost variation across health posts

Supplementary Table S6. Factors associated with average costs

REFERENCES

Supplementary Table S1. SMC costs in context

This table compares the overall costs of SMC and individual incentive payments to relevant expenditure levels or local costs.

SMC cost category	SMC cost		Comparator		Ratio of SMC cost: Comparator
**Financial cost of SMC (excluding research participation incentives) per capita**	Financial cost of SMC (excluding research participation incentives) per capita	$0.32	General government expenditure on health per capita in Senegal in 2014 (World Health Organization, 2017)	$26	1.2%
	Financial cost of SMC (excluding research participation incentives) per capita	$0.32	Total health expenditure per capita in Senegal in 2014 (World Health Organization, 2017)	$50	0.6%
	Financial cost of SMC (excluding research participation incentives) per capita	$0.32	Average annual expenditure for malaria control and elimination per capita in Senegal in 2013-15 (includes both domestic expenditure on malaria prevention and treatment and donor funding earmarked for malaria control) (WHO Global Malaria Programme., 2016)	$2.59	12.4%
**Incentive payments for SMC administration**	CHW daily per diem (mean)	$7.73	Daily wage for unskilled labour	$4.04	193.6%
	Head nurse incentive payments for SMC administration	$242	Head nurse mean annual net salary	$5,894.69	4.1%
	Assistant nurse incentive payments for SMC administration	$121	Assistant nurse mean annual net salary	Not available	NA
**Research participation incentives**	Head nurse (Total per year per person)	$404	Head nurse mean annual net salary	$5,894.69	6.9%
	District Medical Officer (Total per year per person)[2]	$1,818	District medical officer mean annual net salary	$12,000.00	15.2%
	Deputy District Medical Officer (Total per year per person)[2]	$1,212	Deputy District Medical Officer mean annual net salary	$11,176.77	10.8%
	District Supervisor (Total per year per person)[2]	$889	District Supervisor mean annual net salary	$6,048.68	14.7%
	Regional Medical Officer (Total per year per person)	$1,818	Regional Medical Officer mean annual net salary	$12,000.00	15.2%

Supplementary Table S2. Input costs of key cost drivers

This table provides detailed data on the articles (items and payments) that make up the largest proportions of the overall costs of the intervention. For each article, the following is presented: the cost of one unit of the article, the total quantity of the article used in the intervention, the unit measure (e.g. tablets, nurses), and the percentage of total costs of the intervention attributable to that article.

Category	Article	Unit costs (USD)	Total quantity		% Total financial cost (excluding research incentives)	% Total financial cost (including research incentives)
SMC Drugs	Sulphadoxine-pyrimethamine (SP)	$0.02	584,210	Tablets	6.8%	5.5%
	Amodiaquine (AQ)	$0.02	1,837,606	Tablets	21.0%	17.1%
Incentive payments for SMC administration	CHW per diem (mean)	$7.82	10,345	CHW-days	41.4%	33.7%
	CHW per diems received for one month of SMC administration (mean)	$32.41	2497	CHW-months[1]	41.4%	33.7%
	Head nurse SMC incentive payments (total per year per nurse)	$242	46	Nurses	5.8%	4.8%
	Assistant nurse SMC incentive payments (total per year per nurse)	$121	46	Assistant nurses	2.9%	2.4%
Funds provided for fuel costs for supervision	District payments (total per year per district)	$585	4	Districts	1.2%	1.0%
	Prefecture payments (total per year per prefecture)	$390	4	Prefectures	0.8%	0.7%
Research participation incentives	Health Post / Head nurse (Total per year per nurse)	$404	45	Nurses	NA	9.5%
	District (Total per year per district)[2]	$5,697	4	Districts	NA	10.4%
	District Medical Officer (Total per year)[3]	$1,818	4	DMOs	NA	3.7%
	Deputy District Medical Officer (Total per year per district)[4]	$1,212	3	DDMOs	NA	1.9%
	District Supervisor (Total per year per district)[5]	$889	9	Supervisors	NA	4.7%
	Region / Regional Medical Officer (Total per year per RMO)	$1,818	3	RMOs	NA	2.9%

Notes: 1) "CHW-months" of administration refers to the period of 1-6 days within a month spent delivering SMC. 2) Unit cost refers to three of four districts. One district received a smaller incentive payment of $2,697 (or 1,335,000 XOF). 3) Unit cost refers to three of four districts. One DMO received 11 rather than 12 months' payments. 4) One DDMO did not receive payments. 5) One district's supervisors did not receive payments.

Supplementary Table S3. Resources used in SMC delivery

		Number or mean	Health post range		
			s.d.	Low	High
Health structures	Regions	3	NA	NA	NA
	Districts	4	NA	NA	NA
	Health posts[1]	46	NA	NA	NA
Health workers	Head nurses	46	NA	NA	NA
	Assistant nurses[2]	46	NA	NA	NA
	CHWs administering SMC each month (mean)	831.0	NA	NA	NA
	CHWs administering SMC each month per health post	18.3	14.1	4.0	70.0
	Number of days worked on SMC administration per month per CHW	4.2	0.6	1.0	6.0
	Average number of hours worked on SMC per day per CHW (health post mean)	7.4	1.1	4.2	10.0
	Number of hours worked on SMC per day per CHW (individual CHW)	7.2	1.8	1.0	12.0
Outputs per structure or worker	SMC courses administered each month per health post (mean)	3415.1	2,749.7	502.0	16,720.0
	Average number of SMC courses administered per CHW per day (health post mean)	46.0	10.4	25.1	77.5
	Average number of SMC courses administered per CHW per month (health post mean)	190.0	36.5	104.5	272.7
	SMC courses administered each month per CHW (individual CHW)	195.8	147.1	7.0	677.5
	SMC courses administered per CHW per day (individual CHW)	49.4	74.6	1.8	169.4

Supplementary Table S4. Variation in health worker time spent on SMC by month and catchment area

The table shows the cumulative number of hours worked at each health post and at each district and how these varied across health posts and districts. *While the demographic surveillance system staff are primarily employed to carry out research activities, the time presented here represents their contribution to the implementation of SMC, rather than research activities.

Level	Role	Cumulative hours at each health post or district over the season				Distribution of cumulative mean hours spent on SMC across months		
		min	median	Mean	max	Sept	Oct	Nov
**Health Post**	Head nurse	7	75	80	156	46	17	16
	Assistant head nurse	0	48	48	120	23	14	11
	CHWs (Relais)	331	1555	1751	5740	665	543	543
	CHWs (ASC)	0	1	10	109	6	3	1
**Districts**	District medical officer	12	75	60	80	35	10	15
	Deputy district medical officer	0	13	39	132	14	6	19
	District Supervisor	42	206	208	376	113	53	42
	Demographic surveillance system supervisor*	44	73	82	137	44	38	0
	Demographic surveillance system fieldworker*	35	123	148	309	71	77	0

Supplementary Table S5. Descriptive statistics: Cost variation across health posts
HP: Health post. S.D.: standard deviation.

**Variable**		**Obs**	**Mean**	**SD**	**Min**	**Max**
Costs by health post (District costs allocated equally across health posts within each district and research participation incentives included)	AVERAGE Economic Costs, US Cents	46	76.75	36.09	31.93	210.42
	TOTAL Economic Costs, USD	46	6,064	2,540	3,223	15,946
	Log(AVERAGE Economic Costs, US Cents)	46	1.85	0.17	1.50	2.32
Output quantity (i.e. scale)	Courses of SMC administered	46	10,245	8,205	1,562	49,941
	Log(Courses)	46	3.90	0.31	3.19	4.70
Coverage	Coverage (number of courses administered as % of target)	46	0.82	0.14	0.52	1.16
Prior experience	Years of experience with SMC at health post	46	1.80	0.74	1.00	3.00
	Years of experience with SMC of head nurse	46	1.70	0.78	1.00	3.00
Health post geography	Number of villages in health post catchment	46	25.22	19.53	1.00	78.00
	Catchment area, square kilometers	46	25.65	32.33	0.00	126.97
	Minimum from HP to nearest catchment village, kilometers	46	0.17	0.20	0.01	1.08
	Mean from HP to catchment villages, kilometers	46	2.51	1.48	0.16	5.83
	Maximum from HP to furthest catchment village, kilometers	46	5.34	3.16	0.19	14.26

Supplementary Table S6. Factors associated with average costs

The factors associated with variation in average cost per course administered between health posts were explored using linear regression with fixed effects at the district level, as follows: $\boldsymbol{Log(AC_{ij}) = \alpha_i + x'_{ij}\beta + e_{ij}}$, where: AC: average economic cost of SMC; *i*: health posts; *j*: districts; α: district dummy variable; *x'*: covariate vector; β: coefficient on each covariate; e_{ij}: error, independent and normally distributed. Standard regression diagnostics were performed to check for unusual and influential data, normality of residuals, heteroscedasticity, multicollinearity, non-linearity, and model specification error. (Chen et al., 2003) All independent variables were centred. ***p<0.0032, **p<0.01, *p<0.05

	**Estimates of coefficients (95% confidence intervals) for log_{10} (average economic costs)**	
**Parameters**	**Model 1: Complex model with interaction terms**	**Model 2: Parsimonious model**
**Log_{10} (Number of courses)**	-0.498 (-0.543, -0.452)***	-1.65 (-2.53,-0.76)***
**$(Log_{10}$ (Number of courses)$)^2$**	0.339 (0.201, 0.478)***	0.171 (0.058,0.285)***
**Size of catchment area (km^2)**	0.0005 (-0.0001, 0.0010)	-
**Coverage (%)**	0.092 (-0.012, 0.196)	-
**Log_{10} (Number of courses) x Size of catchment area (km^2)**	-0.002 (-0.003, -0.00009)*	-
**Size of catchment area (km^2) x Coverage (%)**	0.005 (0.001, 0.008)**	-
**District:**		-
**District 1**	Reference	Reference
**District 2**	-0.002 (-0.038, 0.033)	-0.018 (-0.063,0.027)
**District 3**	-0.016 (-0.058, 0.027)	0.009 (-0.032,0.050)
**District 4**	-0.032 (-0.067, 0.002)	0.000 (-0.049,0.049)
**Constant**	1.841 (1.815, 1.867)***	1.837 (1.812, 1.861)***
**No. of observations**	46	46
**R^2**	0.962	0.950
**Adjusted R^2**	0.952	0.944

REFERENCES

CHEN, X., ENDER, P., MITCHELL, M. & WELLS, C. 2003. Regression diagnostics. *Regression with Stata*. http://stats.idre.ucla.edu/stata/webbooks/reg/chapter2/stata-webbooksregressionwith-statachapter-2-regression-diagnostics/.

WHO GLOBAL MALARIA PROGRAMME. 2016. World malaria report 2016. Geneva, Switzerland: World Health Organization.

WORLD HEALTH ORGANIZATION 2017. Global Health Expenditure Database. Geneva: WHO.
